# Comparison of visual assessment and computer image analysis of intracoronary thrombus type by optical coherence tomography

**DOI:** 10.1371/journal.pone.0209110

**Published:** 2018-12-17

**Authors:** Timo P. Kaivosoja, Shengnan Liu, Jouke Dijkstra, Heini Huhtala, Tej Sheth, Olli A. Kajander

**Affiliations:** 1 Heart Hospital, Tampere University Hospital and Faculty of Medicine and Life Sciences, University of Tampere, Tampere, Finland; 2 Division of Image Processing, Department of Radiology, Leiden University Medical Center, Leiden, Netherlands; 3 Faculty of Social Sciences, University of Tampere, Tampere, Finland; 4 McMaster University and Population Health Research Institute, Hamilton Health Sciences, Hamilton, Canada; Campus Bio-Medico University of Rome, ITALY

## Abstract

**Background:**

Analysis of intracoronary thrombus type by optical coherence tomography (OCT) imaging is highly subjective. We aimed to compare a newly developed image analysis method to subjective visual classification of thrombus type identified by OCT.

**Methods:**

Thirty patients with acute ST elevation myocardial infarction were included. Thrombus type visually classified by two independent readers was compared with analysis using QCU-CMS software.

**Results:**

Repeatability of the computer-based measurements was good. By using a ROC, area under curve values for discrimination of white and red thrombi were 0.92 (95% confidence intervals (CI) 0.83–1.00) for median attenuation, 0.96 (95% CI 0.89–1.00) for mean backscatter and 0.96 (95% CI 0.89–1.00) for mean grayscale intensity. Median attenuation of 0.57 mm^-1^ (sensitivity 100%, specificity 71%), mean backscatter of 5.35 (sensitivity 92%, specificity 94%) and mean grayscale intensity of 120.1 (sensitivity 85%, specificity 100%) were identified as the best cut-off values to differentiate between red and white thrombi.

**Conclusions:**

Attenuation, backscatter and grayscale intensity of thrombi in OCT images differentiated red and white thrombi with high sensitivity and specificity. Measurement of these continuous parameters can be used as a less user-dependent method to characterize *in vivo* thrombi. The clinical significance of these findings needs to be tested in further studies.

## Introduction

Intracoronary thrombus is a frequent finding in patients with acute coronary syndromes undergoing invasive angiography and percutaneous coronary intervention (PCI). Research on the clinical significance of thrombus type is ongoing, and with more detailed understanding of the thrombotic process, the goal is to facilitate the development of tailored therapies for patients with acute coronary syndromes. Optical coherence tomography (OCT) is a high-resolution intravascular imaging technique with excellent contrast between the vessel lumen and intravascular structures [1]. In a consensus paper, the evidence level for image interpretation of intracoronary thrombus using OCT has been considered to be high [2]. The ability of OCT to produce images allowing differentiation between thrombus types has been validated against histology in post-mortem samples of human coronary arteries [3]. It is widely accepted in the OCT community that thrombi in OCT images can be classified as high-backscattering with a signal-free shadowing (‘red’, erythrocyte-rich) or low-backscattering (‘white’, thrombocyte-rich) [2]. This encourages further validation in clinical patients. However, valid analysis of different thrombus types *in vivo* by OCT is hindered by the high degree of subjectivity of measurement and the lack of validated methodology.

The aim of the present study is to develop a computer image analysis -based method for the assessment of thrombus morphology in OCT images that can be used for further *in vivo* validation. Computer algorithms determining backscatter, attenuation and intensity of the OCT signal are tested and compared to the present standard method, which is consensus classification of thrombus type by two independent analysts. Our hypothesis is that applying computer image analysis to assess thrombus type is feasible and can reduce observer variation in comparison to subjective evaluation.

## Patients and methods

### Patients

The study was approved by the Ethical board at Pirkanmaa Hospital District, approval number R10131. Written informed consent was obtained from all participants. The studied series included 30 patients enrolled in an OCT substudy of the TOTAL trial. The TOTAL trial was an international, multicentre, randomized trial of routine thrombectomy (using the Export catheter, Medtronic Cardiovascular, Santa Rosa, CA, USA) compared with PCI alone in STEMI patients treated with PCI (n = 10732) [4,5]. A prospective OCT sub-study (n = 214) of TOTAL trial evaluated thrombus burden in patients with symptoms of myocardial ischaemia lasting for ≥ 30 min and definite electrocardiographic changes indicating STEMI who were referred for primary PCI and randomized within 12 h of symptom onset [6]. Participation in the study required restoration of TIMI 2–3 flow after the first device. Patients were excluded from the OCT sub-study if they were in cardiogenic shock or had known renal failure. Informed consent was obtained from all individual participants included in the study. Of the patients in the OCT substudy who were randomized to thrombectomy, 72 had good quality OCT pullbacks available preceding balloon predilatation. Out of these, 30 patients with the largest maximal thrombus areas were selected for the present study (for detailed patient flow-chart, see S1 Fig). All procedures performed in studies involving human participants were in accordance with the ethical standards of the institutional and/or national research committee and with the 1964 Helsinki declaration and its later amendments or comparable ethical standards. This article does not contain any studies with animals performed by any of the authors.

### OCT imaging

OCT imaging was performed using the Ilumien OCT system and C7 Dragonfly catheter (St Jude Medical, Minnesota, MN, USA). The radio-opaque distal marker of the OCT catheter was positioned 1–2 cm distal to the target lesion or stent and contrast was injected either manually or by automatic injector, according to local practice. Adequate image quality was checked and additional images were obtained, if the image quality was sub-optimal. OCT imaging raw data was exported in digital format for off-line analysis.

### Optical coherence tomography image analysis

Data on thrombus cross-sectional areas at the culprit lesion was obtained from previous analysis (6). For each patient, three OCT cross-sections with the largest thrombus areas were analyzed.

### Visual assessment of thrombus type

Visual thrombus characteristics were evaluated by two independent readers (T.P.K. and O.A.K.) to assess the inter-observer reproducibility. One observer (T.P.K.) repeated the analysis after > 4 week period to assess the intra-observer reproducibility. Thrombus was graded by a six-stage scale, where value 1 represents purely white thrombus and value 6 purely red thrombus, creating thrombus attenuation score (TAS). In accordance to published standards [1,2], thrombus was considered white or mostly white, when the abluminal border of the thrombus or the vessel wall behind it could be seen. Conversely, the more the thrombus caused shadowing and poor visibility of the structures behind it, the redder it was scored. In addition, we applied only two additional rules. If a cross-section contained multiple fragments with different visual appearances, TAS was defined for the largest fragment. In the case of multiple same-sized thrombi, TAS was defined for the most strongly attenuating part of the thrombus.

### Computer image analysis of thrombus

**Fig 1:**
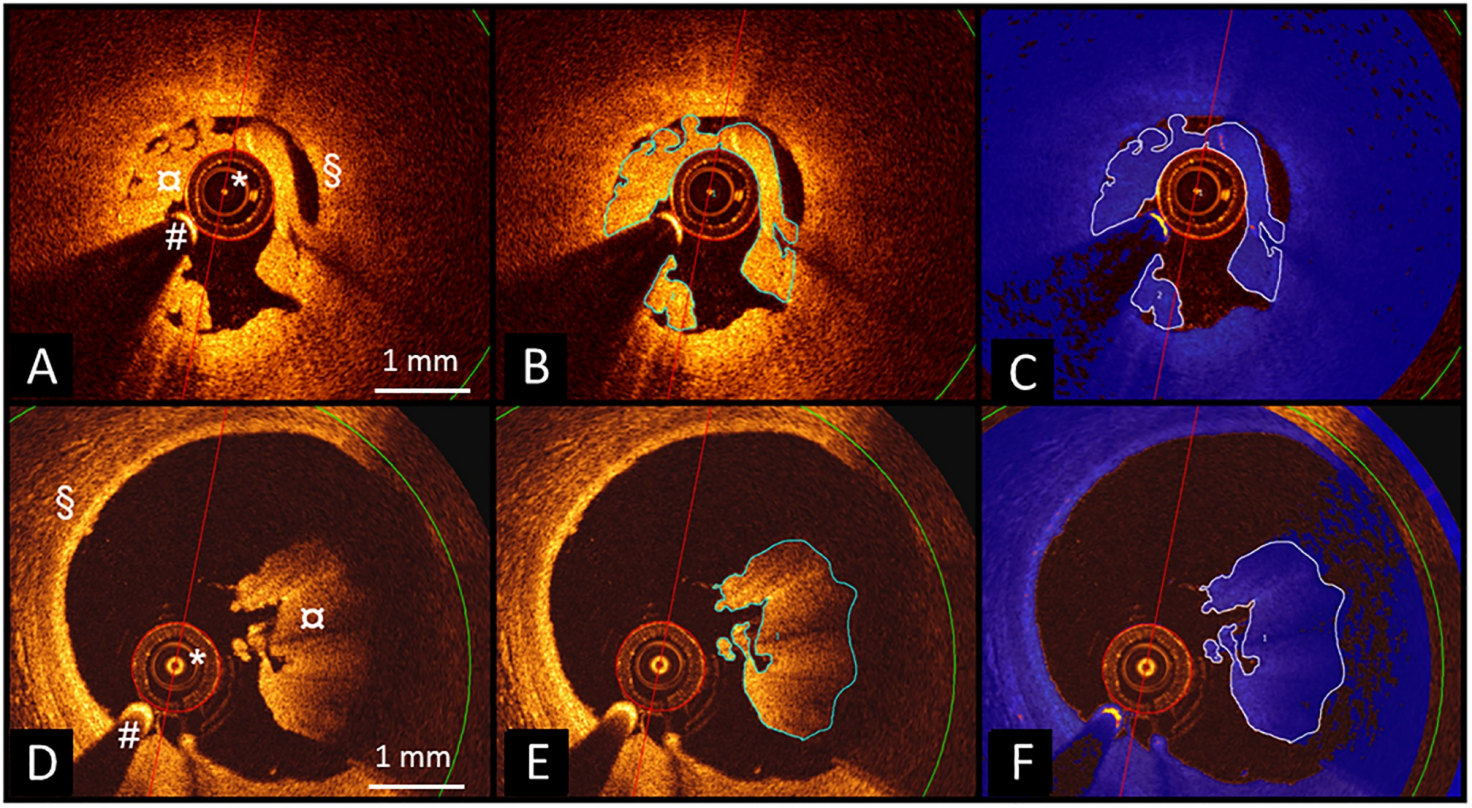
Examples of thrombus assessment by the QCU-CMS image analysis software in OCT image frames of two patients with white (A) and red thrombus (D). After manual tracing of the thrombi (B,E), attenuation analysis was performed including regions with attenuation values above the designated threshold value (displayed in blue) (C,F). Accurate segmentation of the luminal border of the thrombus and the contrast-filled flow area of the vessel can be seen in C and F. *) OCT catheter, #) guidewire artefact, ¤) thrombus, §) vessel wall. OCT, optical coherence tomography.

A special version of QCU-CMS version 4.69 software (Leiden University Medical Center, Leiden, The Netherlands) was used by two independent analysts. First, the thrombus areas were manually traced. The software then determined attenuation coefficient, backscatter term and grayscale intensity values for these areas and calculated several statistical parameters, including median, mean, standard deviation and 5^th^, 10^th^, 90^th^ and 95^th^ percentile values. Two patient cases with white and red thrombus showing the method are shown in Fig 1. To avoid the inclusion of noise pixels and dark areas (low attenuation) at the abluminal side of the thrombus, we experimented with different minimum threshold values for attenuation. For the present analyses, attenuation was analyzed using both 0.15 mm^-1^ and 0.5 mm^-1^ threshold values.

The light attenuation coefficient and backscatter term have been determined based on a “depth-resolved” model [7]. The attenuation coefficient describes the total decreasing rate of light when it is traveling though the tissue. OCT images are generated by receiving the backscattered light. The backscatter coefficient describes the efficiency of tissue scattering light backwards. The backscatter term is estimated to be related to the backscatter coefficient. More information about the estimation can be referred to the technical paper [7]. The advantage of this method is its fast pixel-wise estimation, which requires no predefined delineation and is more flexible for post analysis.

For each patient, a weighted average of each statistical parameter was calculated using the pixel count in each traced thrombus area. To study inter-observer reproducibility, two readers analyzed the same OCT cross-sections of 10 randomly selected patients independently. For intra-observer comparison, one observer repeated the analyses on these 10 patients after a 4-week period. For the repeated assessments, OCT raw data was re-imported into the QCU-CMS software. Then the thrombus areas were manually re-traced in the same transversal frames.

### Statistical analysis

Bland–Altman (B-A) analysis was used to estimate the bias resulting from inter- and intra-observer variation [8]. In the analysis, the mean values of observer 1 and 2 were plotted against the difference of measurements between the observers. The 95% limits of agreement (equal to ±1.96 SD) were determined for the variables. Mean relative difference for each variable was defined as the mean absolute difference divided by mean of observers 1 and 2. Intraclass correlation coefficients (ICCs) and their 95% confidence intervals were calculated based on absolute-agreement, 2-way mixed effect model. Spearman correlations were calculated between the continuous variables and TAS. Inter- and intraobserver reliability of thrombus classification was assessed using the kappa statistic (κ). Receiver-operating characteristic (ROC) curve analyses were performed to determine the best cutoff values (using highest Youden Index) of QCU-CMS parameters for discriminating red and white thrombi and the areas under the curve (AUCs), as well as the sensitivities and specificities of the diagnostic test. In addition, binary logistic regression analysis was used to evaluate how the different continuous image analysis software parameters predicted the type of thrombus. Statistical analyses were performed with the Stata Statistical software: Release 13 (StataCorp, College Station, TX, USA) and the IBM SPSS 21 Statistics software (IBM, Armonk, NY, USA).

## Results

Baseline patient characteristics are shown in Table 1.

**Table 1:** Baseline patient characteristics.

N = 30	
Age, years (mean ± SD)	57 ± 10.1
Male sex, N (%)	22 (73.3)
Risk factor profile, N (%)	
Hypertension	14 (46.6)
Diabetes	4 (13.3)
Current smoking	8 (26.7)
Obesity (BMI ≥ 30)	14 (46.7)
Prior MI	2 (6.7)
Prior PCI	1 (3.3)
Creatinine, μmol/L (mean ± SD)	82.0 ± 16.6
Antithrombotic treatment, N (%)	
Upfront glycoprotein inhibitor IIb/IIIa use	11 (36.7)
UFH prior to procedure	22 (73.3)
Enoxaparin prior to procedure	2 (6.7)
Bivalirudin prior to procedure	0 (0.0)
Culprit vessel, N (%)	
RCA	16 (53.3)
LAD	12 (40.0)
LCx	2 (6.7)
TIMI flow*, N (%)	
0	15 (50.0)
1	4 (13.3)
2	3 (10.0)
3	8 (26.7)
TIMI thrombus class*, N (%)	
< 3	4 (13.3)
≥ 3	26 (86.7)

* prior to thrombectomy.

BMI, body mass index; LAD, left anterior descending coronary artery; LCx, left circumflex coronary artery; MI, myocardial infarction; PCI, percutaneous coronary intervention; RCA, right coronary artery; SD, standard deviation; TIMI, thrombolysis in myocardial infarction; UFH, unfractionated heparin.

### Qualitative (visual) thrombus classification by two observers

For the purpose of statistical comparison of the thrombus assessment methods, a six-stage scale consensus TAS was used to form a binary classification, where scores 1–3 equaled white and 4–6 red thrombus. There were 13 cases classified as white and 17 as red thrombus (S2 Fig). Kappa values for binary classification of the thrombi were 0.74 and 0.53, respectively, for inter- and intraobserver comparisons.

### Reproducibility of quantitative measurements using QCU-CMS software

**Fig 2:**
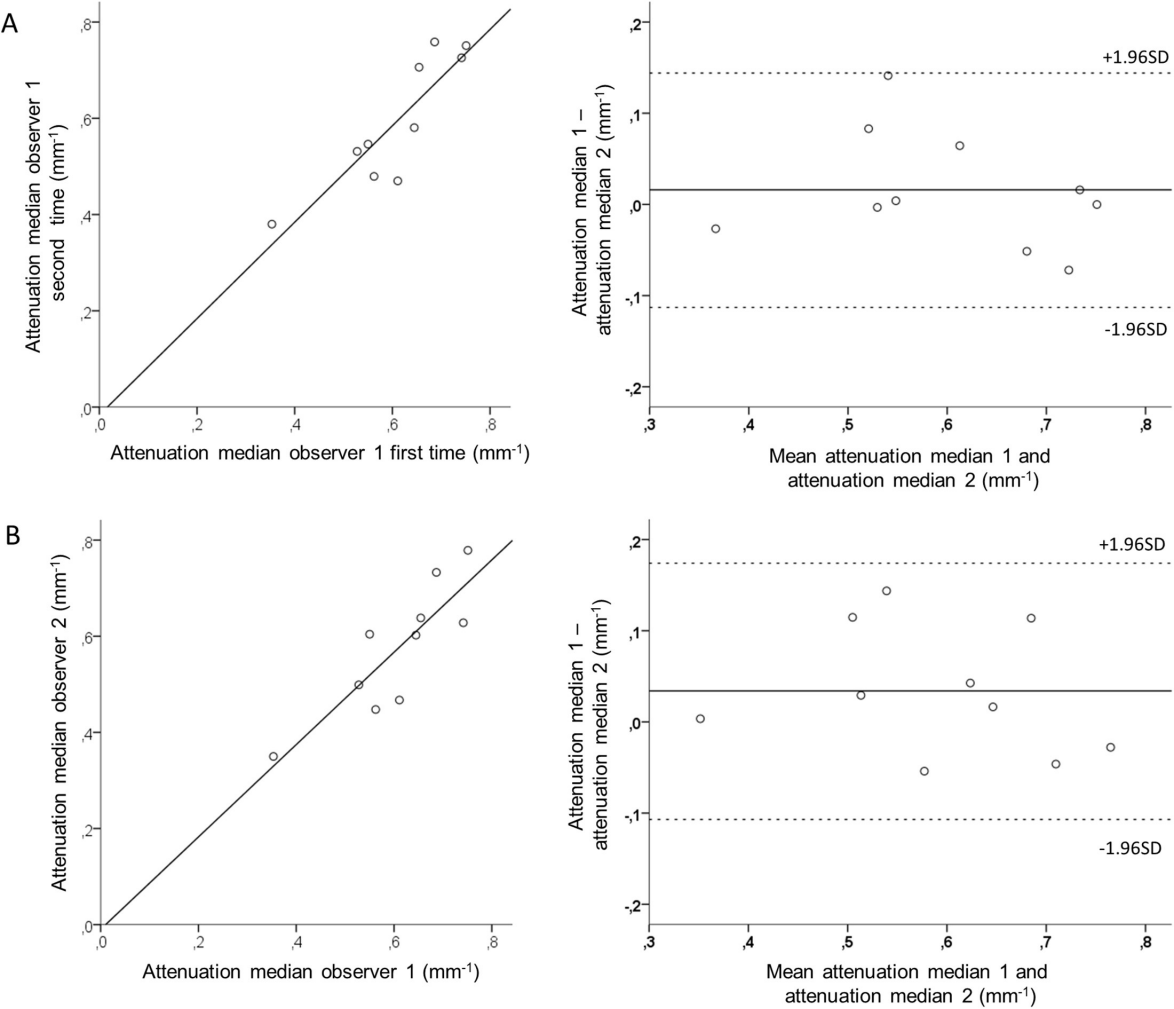
Intra- and interobserver variability of measurement of thrombus attenuation in OCT images using image analysis software. Scatterplot (left) and Bland-Altman plot (right) of intraobserver (A) and interobserver (B) comparison for median attenuation. OCT, optical coherence tomography; SD, standard deviation.

Results presented in Tables 2 and 3 show inter- and intraobserver variability of measurements in thrombus regions. Mean differences for most statistical parameters of thrombus attenuation, backscatter and grayscale intensity were small, with narrow limits of agreement between observers. For all tested parameters, intraobserver variability was slightly smaller than interobserver variation. Limits of agreement for skewness and kurtosis of attenuation, backscatter and grayscale intensity were wider than for the other statistical parameters. B-A plot of median attenuation is shown in Fig 2. B-A plots of mean backscatter and mean grayscale intensity, respectively, are shown in S3 and S4 Figs.

**Table 2:** Interobserver variability of QCU-CMS measurements.

Parameters	Observer 1 Mean (SD)	Observer 2 Mean (SD)	Range	Mean absolute difference (CI)	Mean relative difference (%)	Limits of agreement	ICC (CI)
Attenuation (mm^-1^)							
Median	0.61 (0.12)	0.57 (0.13)	0.35–0.77	0.03 (-0.02–0.08)	5.8	-0.11–0.17	0.91 (0.64–0.98)
Mean	0.72 (0.09)	0.69 (0.11)	0.49–0.83	0.03 (-0.02–0.08)	4.3	-0.10–0.16	0.88 (0.55–0.97)
Standard deviation	0.42 (0.09)	0.42 (0.07)	0.32–0.54	0.001 (-0.02–0.02)	0.2	-0.07–0.07	0.96 (0.84–0.99)
Skewness	1.36 (0.50)	1.46 (0.40)	0.89–2.18	-0.10 (-0.26–0.06)	-7.2	-0.55–0.19	0.93 (0.73–0.98)
Kurtosis	3.01 (2.42)	3.72 (2.45)	0.55–7.21	-0.72 (-2.21–0.78)	-21.4	-4.90–3.47	0.77 (0.14–0.94)
5^th^ percentile	0.26 (0.06)	0.23 (0.06)	0.17–0.38	0.02 (-0.01–0.04)	8.6	-0.04–0.08	0.90 (0.52–0.98)
10^th^ percentile	0.30 (0.07)	0.28 (0.07)	0.19–0.46	0.02 (-0.01–0.05)	8.3	-0.06–0.11	0.90 (0.58–0.97)
90^th^ percentile	1.27 (0.16)	1.24 (0.17)	1.00–1.53	0.03 (-0.03–0.09)	2.3	-0.14–0.20	0.92 (0.71–0.98)
95^th^ percentile	1.51 (0.20)	1.48 (0.19)	1.21–1.77	0.03 (-0.04–0.10)	1.9	-0.17–0.23	0.93 (0.75–0.98)
Backscatter							
Median	5.18 (0.22)	5.09 (0.28)	4.77–5.49	0.10 (-0.01–0.20)	1.9	-0.20–0.40	0.87 (0.46–0.97)
Mean	5.19 (0.18)	5.10 (0.23)	4.79–5.46	0.10 (0.01–0.18)	1.8	-0.15–0.34	0.86 (0.36–0.97)
Standard deviation	0.61 (0.09)	0.66 (0.07)	0.47–0.72	-0.04 (-0.07–-0.02)	-6.9	-0.10–0.01	0.89 (-0.09–0.98)
Skewness	-0.08 (0.29)	-0.22 (0.44)	-0.74–0.31	0.15 (-0.05–0.34)	96.7	-0.39–0.68	0.83 (0.37–0.96)
Kurtosis	-0.13 (0.49)	0.44 (1.24)	-0.70–2.37	-0.57 (-1.16–0.02)	-183	-2.22–1.08	0.70 (-0.05–0.92)
5^th^ percentile	4.22 (0.26)	4.07 (0.26)	3.77–4.70	0.15 (0.05–0.24)	3.5	-0.11–0.41	0.87 (0.05–0.97)
10^th^ percentile	4.41 (0.25)	4.28 (0.28)	3.95–4.93	0.13 (0.04–0.21)	2.9	-0.12–0.37	0.90 (0.26–0.98)
90^th^ percentile	5.99 (0.16)	5.94 (0.18)	5.65–6.26	0.05 (-0.02–0.11)	0.8	-0.14–0.23	0.91 (0.65–0.98)
95^th^ percentile	6.17 (0.16)	6.13 (0.17)	5.85–6.41	0.04 (-0.02–0.10)	0.7	-0.14–0.21	0.92 (0.69–0.98)
Grayscale							
Median	67.19 (32.99)	63.86 (35.43)	28.16–147.83	3.32 (-2.53–9.19)	5.1	-13.05–19.71	0.99 (0.94–1.00)
Mean	104.65 (26.87)	100.04 (31.37)	69.76–166.69	4.61 (-2.28–11.49)	4.5	-14.64–23.85	0.97 (0.88–0.99)
Standard deviation	106.03 (17.46)	103.02 (15.95)	80.03–134.78	3.02 (-3.78–9.81)	2.9	-15.98–22.01	0.91 (0.67–0.98)
Skewness	2.51 (0.88)	2.65 (0.72)	1.72–3.99	-0.14 (-0.41–0.14)	-5.3	-0.91–0.63	0.94 (0.76–0.98)
Kurtosis	12.49 (11.67)	14.62 (10.83)	4.31–38.17	-2.13 (-6.88–2.62)	-15.7	-15.40–11.14	0.91 (0.64–0.98)
5^th^ percentile	16.59 (13.34)	14.74 (11.59)	6.66–48.92	1.85 (0.08–3.61)	11.8	-3.10–6.79	0.99 (0.91–1.00)
10^th^ percentile	21.77 (16.88)	20.14 (16.17)	8.83–65.09	1.63 (-0.02–3.27)	7.8	-2.97–6.22	0.99 (0.96–1.00)
90^th^ percentile	239.64 (40.18)	230.74 (48.77)	166.58–312.48	8.90 (-4.40–22.20)	3.8	-28.28–46.09	0.94 (0.80–0.99)
95^th^ percentile	315.19 (44.98)	305.05 (53.64)	228.63–408.65	10.15 (-6.64–26.94)	3.3	-36.78–57.08	0.94 (0.75–0.98)

N = 10. CI, confidence interval; SD, standard deviation; ICC, intra-class correlation coefficient.

**Table 3:** Intraobserver variability of QCU-CMS measurements.

Parameters	Observer 1 first time Mean (SD)	Observer 1 second time Mean (SD)	Range	Mean absolute difference (CI)	Mean relative difference (%)	Limits of agreement	ICC (CI)
Attenuation (mm^-1^)							
Median	0.61 (0.12)	0.59 (0.13)	0.37–0.75	0.02 (-0.03–0.06)	3.3	-0.11–0.14	0.93 (0.74–0.98)
Mean	0.72 (0.09)	0.70 (0.11)	0.51–0.84	0.02 (-0.02–0.06)	2.8	-0.10–0.13	0.92 (0.69–0.98)
Standard deviation	0.42 (0.09)	0.41 (0.08)	0.31–0.54	0.001 (-0.01–0.02)	0.2	-0.04–0.05	0.98 (0.94–1.00)
Skewness	1.36 (0.50)	1.41 (0.43)	0.75–2.14	-0.05 (-0.24–0.14)	-3.6	-0.58–0.48	0.92 (0.68–0.98)
Kurtosis	3.01 (2.42)	3.64 (2.81)	0.52–6.99	-0.64 (-2.48–1.21)	-19.2	-5.79–4.52	0.69 (-0.24–0.92)
5^th^ percentile	0.26 (0.06)	0.25 (0.06)	0.18–0.39	0.01 (-0.01–0.02)	3.9	-0.03–0.05	0.97 (0.90–0.99)
10^th^ percentile	0.30 (0.07)	0.29 (0.07)	0.20–0.45	0.01 (-0.01–0.03)	3.4	-0.05–0.06	0.97 (0.88–0.99)
90^th^ percentile	1.27 (0.16)	1.25 (0.17)	1.02–1.54	0.02 (-0.03–0.07)	1.6	-0.12–0.16	0.95 (0.82–0.99)
95^th^ percentile	1.51 (0.20)	1.48 (0.21)	1.23–1.79	0.03 (-0.03–0.08)	2.0	-0.13–0.18	0.96 (0.86–0.99)
Backscatter							
Median	5.18 (0.22)	5.13 (0.29)	4.78–5.47	0.05 (-0.06–0.16)	1.0	-0.25–0.35	0.91 (0.65–0.98)
Mean	5.19 (0.18)	5.14 (0.25)	4.80–5.46	0.04 (0.05–0.13)	0.8	-0.20–0.29	0.92 (0.69–0.98)
Standard deviation	0.61 (0.09)	0.62 (0.09)	0.43–0.69	-0.01 (-0.03–-0.01)	-1.6	-0.06–0.04	0.98 (0.92–1.00)
Skewness	-0.08 (0.29)	-0.10 (0.31)	-0.47–0.29	0.02 (-0.10–0.14)	-22.2	-0.32–0.36	0.92 (0.68–0.98)
Kurtosis	-0.13 (0.49)	0.02 (0.56)	-0.76–0.86	-0.15 (-0.44–0.13)	-136	-0.94–0.64	0.83 (0.37–0.96)
5^th^ percentile	4.22 (0.26)	4.16 (0.32)	3.78–4.77	0.05 (-0.04–0.14)	1.2	-0.19–0.29	0.95 (0.82–0.99)
10^th^ percentile	4.41 (0.25)	4.36 (0.31)	3.96–4.94	0.05 (-0.05–0.14)	1.1	-0.21–0.30	0.95 (0.80–0.99)
90^th^ percentile	5.99 (0.16)	5.96 (0.19)	5.66–6.27	0.03 (-0.03–0.09)	0.5	-0.13–0.19	0.94 (0.79–0.99)
95^th^ percentile	6.17 (0.16)	6.14 (0.18)	5.86–6.43	0.03 (-0.02–0.08)	0.5	-0.12–0.32	0.95 (0.81–0.99)
Grayscale							
Median	67.19 (32.99)	70.05 (36.06)	30.67–151.52	-2.86 (-8.00–2.28)	-4.2	-17.23–11.51	0.99 (0.96–1.00)
Mean	104.65 (26.87)	107.50 (29.72)	73.22–170.61	-2.85 (-9.16–3.45)	-1.3	-20.48–14.77	0.98 (0.91–0.99)
Standard deviation	106.03 (17.46)	107.19 (13.59)	91.86–136.54	-1.16 (-7.88–5.56)	-1.1	-19.95–17.64	0.91 (0.63–0.98)
Skewness	2.51 (0.88)	2.52 (0.70)	1.63–3.99	-0.003 (-0.33–0.32)	-0.1	-0.91–0.90	0.92 (0.70–0.98)
Kurtosis	12.49 (11.67)	12.84 (10.82)	3.81–38.65	-0.35 (-5.00–4.30)	-2.8	-13.35–12.65	0.92 (0.66–0.98)
5^th^ percentile	16.59 (13.34)	17.19 (14.43)	7.17–54.29	-0.60 (-1.97–0.76)	-3.6	-4.41–3.21	1.00 (0.98–1.00)
10^th^ percentile	21.77 (16.88)	22.96 (18.30)	9.50–69.53	-1.20 (-2.97–0.58)	-5.4	-6.15–3.76	0.99 (0.98–1.00)
90^th^ percentile	239.64 (40.18)	244.76 (38.86)	195.65–319.26	-5.12 (-19.99–9.75)	-2.1	-46.69–36.46	0.93 (0.73–0.98)
95^th^ percentile	315.19 (44.98)	320.95 (41.95)	273.50–414.96	-5.76 (-24.41–12.90)	-1.8	-57.91–46.39	0.91 (0.63–0.98)

N = 10. CI, confidence interval; SD, standard deviation; ICC, intra-class correlation coefficient.

### Comparison of visual TAS and measurements by QCU-CMS software

**Fig 3:**
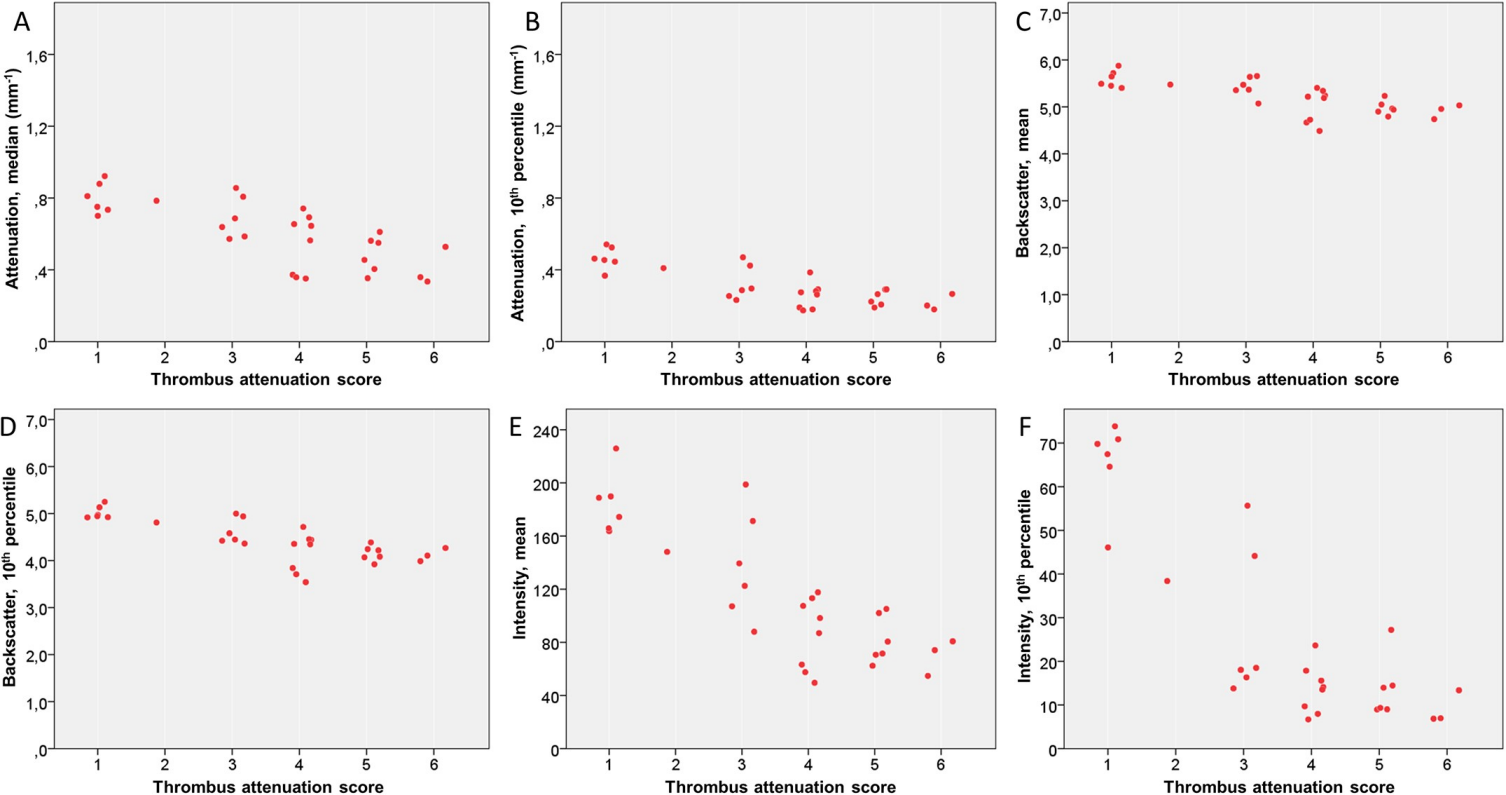
Relationship of thrombus attenuation score and parameters measured by image analysis software in thrombus areas in OCT images. Scatterplots for median attenuation (A), 10^th^ percentile of attenuation (B), mean backscatter (C), 10^th^ percentile of backscatter (D), mean grayscale intensity (E) and 10^th^ percentile of grayscale intensity (F). OCT, optical coherence tomography.

There was an inverse relationship between several QCU-CMS parameters of attenuation (median values, r = -0.79, p<0.001); 10^th^ percentile values r = -0.71, p<0.001), backscatter (mean values r = -0.78, p<0.001; 10^th^ percentile value r = -0.79, p<0.001) and grayscale intensity (mean values r = -0.80, p<0.001; 10^th^ percentile values r = -0-80, p<0.001)) and the visual TAS. Distribution of selected statistical parameters of attenuation, backscatter and grayscale intensity in the six-stage TAS is shown (Fig 3). For the distribution of the parameters using two-stage TAS, see S5 Fig. In these analyses, a minimum threshold 0.15 mm^-1^ was used for attenuation.

The same thrombus cross-sectional areas were analyzed using a higher minimum threshold (0.5 mm^-1^) for attenuation. This resulted in exclusion of an average of 36% (minimum 5.3%, maximum 69.8%) of the pixels of each patient previously included with the 0.15 mm^-1^ threshold. In this analysis, only low percentiles of attenuation values (for 10^th^ percentile values, r = 0.59, p<0.001) had an inverse relationship with visual TAS. Median (r = -0.05, p = 0.78) or 95^th^ percentile values (r = 0.34, p = 0.07) of attenuation values were not statistically significantly related to TAS (S6 Fig).

In addition, using ratio of attenuation and intensity measurements, a new ‘normalized’ variable was constructed. The ratio of 95^th^ percentile of attenuation and median intensity had a direct relationship with TAS (r = 0.86, p<0.001) (S7 Fig).

### ROC analysis

**Fig 4:**
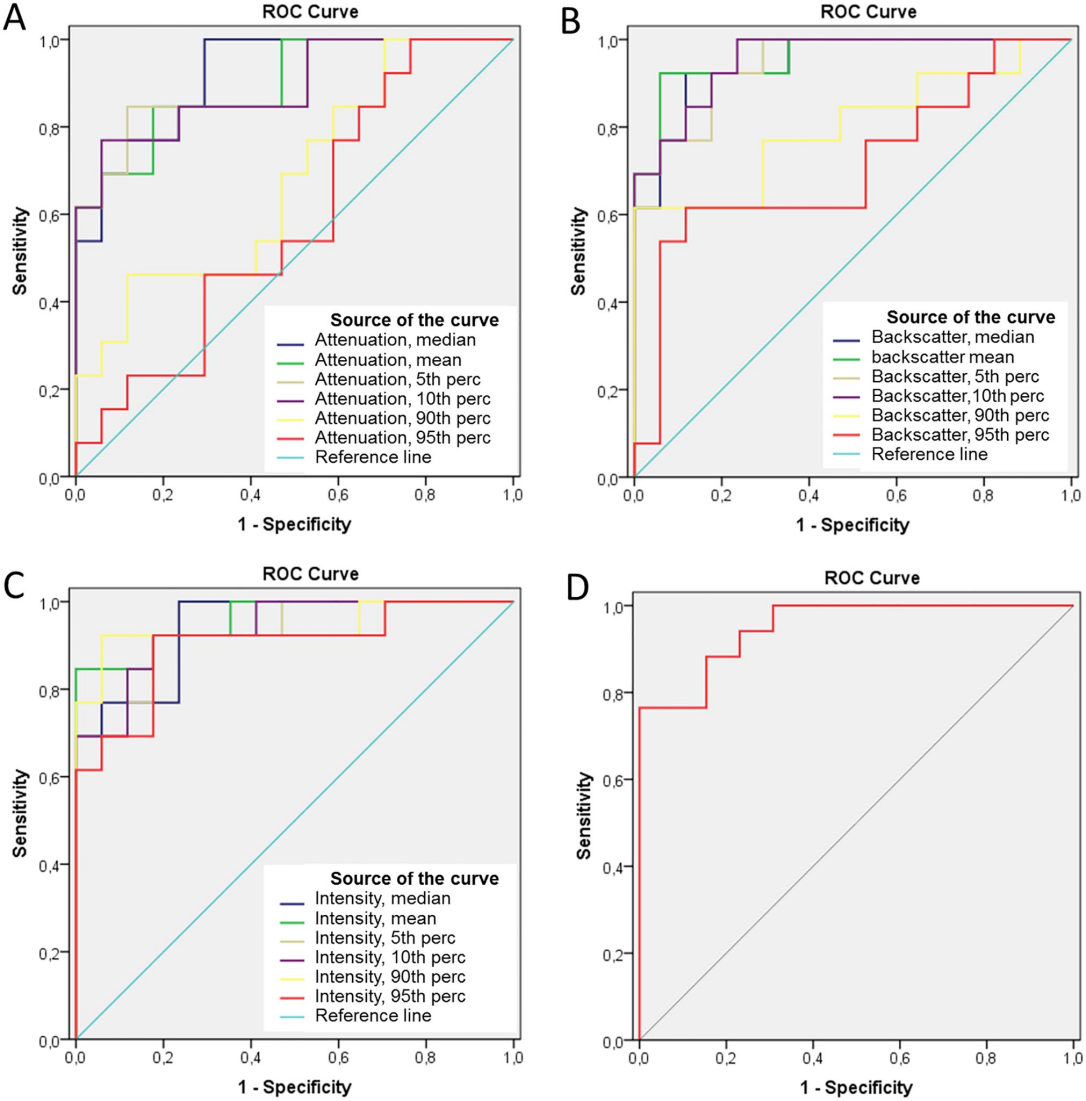
Receiver operating characteristic (ROC) curves of parameters determined by QCU-CMS software in prediction of thrombus type (white vs. red). Different statistical parameters of attenuation (A), backscatter (B), grayscale intensity (C) and the ratio of 95^th^ percentile of attenuation and median grayscale intensity (D). Perc, percentile.

A ROC curve analysis was applied to assess the best parameters to distinguish between different types of thrombus. The AUCs represented the ability of QCU-CMS software parameters to differentiate red and white thrombi (Table 4). The best cutoff values to diagnose red thrombus in each class of variables were <5.35 for mean backscatter (AUC = 0.959, sensitivity 92%, specificity 94%), <120.1 for mean grayscale intensity (AUC = 0.959, sensitivity 85%, specificity 100%), <0.57 mm^-1^ for median attenuation (AUC = 0.919, sensitivity 100%, specificity 71%) and >0.022 mm^-1^ for 95^th^ percentile of attenuation/median grayscale intensity (AUC = 0.950, sensitivity 88%, specificity 85%) (Fig 4). In an additional analysis, using binary logistic regression, best predictors of thrombus type were unchanged.

**Table 4:** Results of receiver operating characteristic (ROC) curve analysis of values determined by QCU-CMS software in prediction of thrombus type (white vs. red).

Variable	AUC	95% CI	Sens (%)	Spec (%)	CP
Attenuation (mm^-1^)					
Median	0.919	0.83–1.00	100	71	0.568
Mean	0.896	0.78–1.00	85	82	0.753
5^th^ percentile	0.896	0.78–1.00	85	88	0.246
10^th^ percentile	0.891	0.77–1.00	77	94	0.294
90^th^ percentile	0.679	0.48–0.87	46	88	1.384
95^th^ percentile	0.584	0.38–0.79	100	24	1.222
Standard deviation	0.624	0.42–0.83	85	18	0.322
Skewness	0.186	0.02–0.35	85	12	0.778
Kurtosis	0.312	0.11–0.52	85	12	0.849
Backscatter					
Median	0.946	0.87–1.00	92	88	5.33
Mean	0.959	0.89–1.00	92	94	5.35
5^th^ percentile	0.946	0.87–1.00	92	82	4.19
10^th^ percentile	0.955	0.89–1.00	100	77	4.36
90^th^ percentile	0.801	0.63–0.97	62	100	6.20
95^th^ percentile	0.710	0.51–0.91	62	88	6.33
Standard deviation	0.837	0.67–1.00	15	77	0.694
Skewness	0.072	0.00–0.17	62	6	-0.417
Kurtosis	0.882	0.76–1.00	69	100	0.110
Grayscale intensity					
Median	0.941	0.86–1.00	100	77	57.11
Mean	0.959	0.89–1.00	85	100	120.1
5^th^ percentile	0.928	0.84–1.00	92	82	12.03
10^th^ percentile	0.937	0.85–1.00	92	82	15.95
90^th^ percentile	0.941	0.84–1.00	92	94	261.6
95^th^ percentile	0.900	0.78–1.00	92	82	337.6
Standard deviation	0.756	0.58–0.94	69	77	106.7
Skewness	0.081	0.00–0.18	8	53	2.72
Kurtosis	0.100	0.00–0.23	8	82	14.86
95^th^ percentile of attenuation/median intensity (mm^-1^)	0.950	0.88–1.00	88	85	0.022

N = 30. AUC, area under curve; CI, confidence interval; CP, cut-off point defined by the largest value of J (Youden) index; Sens, sensitivity; Spec, specificity.

AUCs for different statistical variables of attenuation measurements from the second analysis using a higher 0.5 mm^-1^ threshold were low (in range of 0.50–0.60) except for 5^th^ percentile and 10^th^ percentile of attenuation (AUCs 0.878 and 0.851, respectively).

## Discussion

As a first step in order to resolve the clinical significance and predictive potential of OCT-based thrombus analysis in clinical patients, a robust method to translate the traditional visual classification into reproducibly measured variables is needed. Validated methodology to assess different thrombus types by OCT imaging has not been available, and previously published data has relied on subjective analysis methods based on observers’ visual perception of the thrombus type [9,10]. In this methodological study, we measured attenuation, backscatter and grayscale intensity of *in vivo* thrombi using newly developed image analysis software features. For methodological validation, we assessed repeatability of the measurements and the correlation between traditional visual thrombus classification and the computer software-based analysis. We were able to demonstrate for the first time the use of image analysis software to assess *in vivo* thrombi in OCT images. Measurements of attenuation, backscatter and grayscale intensity of the thrombi using the software were highly repeatable and there was good agreement between two observer consensus visual evaluation of thrombus type and several parameters measured using the software on the OCT images.

Visual assessment of thrombus type in OCT images is *per se* highly observer-dependent. There is not much previous data on the repeatability of the operator-based assessment of thrombus type and there is also a lack of standardized definitions and cut-off values differentiating red and white thrombus. We observed that the visual classification, as expected, was only moderately reproducible. Of note, the present analysis was performed off-line by experienced analysts and reproducibility outside the OCT core lab setting is most probably even lower. In the present study, we showed that computer-based image analysis of *in vivo* thrombus can be performed with good repeatability. The OCT-derived mean and 10^th^ percentile backscatter and mean grayscale intensity values and the ratio of 95^th^ percentile of attenuation to median grayscale intensity had excellent performance (AUC >0.95) in discriminating red from white thrombus. In addition, median values of attenuation, backscatter and grayscale intensity, low percentile values of the same variables and high percentile values of grayscale intensity all had good performance (AUC>0.90).

Earlier histopathological studies of aspirated coronary thrombi have provided important insight to the composition of the thrombus [11,12]. Specimens retrieved by aspiration devices, however, do not necessarily represent the whole thrombotic burden in a patient with STEMI. Some of the thrombotic material stays attached to the vessel wall and may differ from the aspirate. Further, the structure of thrombus is easily distorted and the material fragmented during aspiration. A thorough analysis of aspirated thrombus fragments would be highly laborious and as such subject to a variety of sources of error. The three-dimensional structure of the in vivo thrombus is lost and the co-localization of thrombus and the culprit lesion is not possible. The ability of intravascular OCT to provide high-resolution images of thrombus i*n vivo* during angiography and PCI procedures [13] makes it a robust tool to study thrombus. A further advantage of OCT imaging in comparison to histological analysis of thrombus aspirate is that the images are available for intraoperative decision-making. Considering the frequent occurrence of thrombus in OCT imaging of clinical patients, especially in case of acute coronary syndromes, the data on thrombus image analysis is surprisingly scarce. In a single cadaver series, OCT light attenuation characteristics have been validated against histology to classify thrombi as red or white. When the authors applied a cut-off value defined in that study, OCT had good sensitivity and specificity for differentiating red and white thrombi. [3] In addition to a case report [14] the method has not been used successively.

The goal of analyzing thrombus type *in vivo* and gaining insight to the pathogenesis of acute myocardial infarction is to facilitate the development of novel therapies and to tailor the intervention for each patient. There is some evidence that aspirates consisting mainly of white thrombus might be predictive of lower mortality after the infarction [15]. Presence of older thrombus in even 40% of thrombus aspirates of acute STEMI patients in a large series [16] supports the concept that the acute closure of the culprit artery is preceded by days or weeks of thrombotic process. Interestingly, histological signs of older thrombus have been associated with higher mortality after the myocardial infarction [16]. In addition, red thrombus in the aspirate has been suggested to associate with more frequent angiographically visible distal embolization [17]. Development of reliable image analysis methodology is essential to enable investigation of thrombus types in vivo. In the future, development of smaller size imaging catheters may reduce the need for routine thrombus aspiration prior to OCT imaging of highly thrombotic lesions, such as those in STEMI patients.

In a recent *in vitro* study, the correlation between red blood cell concentration and attenuation or backscatter of the OCT signal was shown to have a limit, above which the correlation was not seen [18]. In our series of *in vivo* OCT imaging, we did not observe any saturation threshold level for these variables. A feasible explanation for this might be that the red blood cell concentration achieved in an experimental model is higher than in thrombus *in vivo*, which contains other components as well. Unexpectedly, in the present study we were not able to demonstrate a higher actual attenuation in red than white thrombus using the software algorithm Instead, there was an inverse relationship between attenuation values and red thrombus, including high percentiles of attenuation. Even after setting a higher threshold for minimum attenuation, which removed all ‘dark’ areas in the abluminal side of the thrombus when strong signal attenuation was present, a positive correlation was not observed. This seems to be due to high heterogeneity of analyzed thrombus regions. In red thrombi, there are highly attenuating parts, but these are also observed in the white thrombus.

We tested different parameters measured by the software from the thrombus images. Attenuation is a parameter, which describes the relative decrease in the light intensity per distance travelled in the tissue. This is different from backscatter and grayscale intensity, which both describe the absolute values of light properties. In the present series, we observed that grayscale intensity performed as well as attenuation in differentiating thrombus types. There were also no differences in observer variation between attenuation and grayscale intensity parameters. An explanation for this finding may be the high technical quality of the patient subset selected for the analysis. In presence of more blood speckle i.e. residual artefactual blood in the image frames, attenuation is expected to be a more stable parameter. On the other hand, intraluminal thrombus is in most occasions located very proximally to the imaging catheter. This may render thrombus analysis less vulnerable to artefacts, such as small quantities of artefactual blood in the OCT recording.

The tested image analysis methodology relies on automatic measurement of attenuation and backscatter along the A-lines of the OCT image. The analysis is not automatic, and user-dependent interaction is needed, because the software does not integrate global information of the cross-section, considering artefacts and thrombus-like structures. Although it would be possible to use image analysis algorithms to determine most of the borders of thrombus cross-sections, in most cases some manual correction would be needed, especially for the red thrombus where no abluminal border is visible. For that reason and to simplify the testing of observer variation, in this study we decided to use manual tracing of the thrombus cross-sections. In addition, we applied a minimum threshold value for attenuation, which excludes noise pixels and dark regions accidentally included in the regions of interest. By using this threshold, we also aimed at avoiding inclusion of non-informative pixels in the abluminal side of the thrombus, where OCT signal has attenuated to a minimum. For further development of the methodology, it can be possible to aim for a semi-automatic analysis method, starting from delineation of intraluminal space followed by automatic detection of luminal border of the thrombus with necessary manual corrections.

### Limitations

The current study has a number of important limitations. First, the thrombus regions were manually delineated. To reduce user-dependence, we applied a minimal threshold value to attenuation, which effectively delineated the endoluminal border of the thrombus. The abluminal border of the thrombus area, especially in the case of red thrombi was, however, partly dependent on manual tracing. Second, since this was an *in vivo* study including clinical patients, no histological golden standard could be applied to the ROC curve analyses. This prevents us from establishing a direct correlation between the software-derived parameters and biological thrombus contents. The basic principle of increased light attenuation by increased red blood cell content of the thrombus has, however, been validated earlier [3]. Third, the current study was not designed to assess the real-world reproducibility, but was limited to a relatively small sample of selected cases. We focused on improving the reproducibility of thrombus type analysis by the use of computer-based methodology. More data is needed in order to evaluate the true absolute reproducibility.

## Conclusion

Subjective thrombus type assessment in OCT images can be complemented by less user-dependent computer-based image analysis methodology. Computer-based measurement of attenuation, backscatter and grayscale intensity of thrombi is repeatable and can be used to differentiate red and white thrombi with high sensitivity and specificity. This study stimulates further studies with larger sample size to confirm the results and their clinical value.

## Supporting information

S1 Fig Patient flow chart of the study. OCT, optical coherence tomography.
(TIF)

S2 Fig Qualitative thrombus classification. Consensus classification by two observers is shown. Thrombus attenuation score (TAS) from 1 to 3 equals white thrombus and from 4 to 6 red thrombus.
(TIF)

S3 Fig Intra- and interobserver variability of measurement of thrombus backscatter in OCT images using image analysis software. Scatterplot (left) and Bland-Altman plot (right) of intraobserver (A) and interobserver (B) comparison for mean backscatter. OCT, optical coherence tomography; SD, standard deviation.
(TIF)

S4 Fig Intra- and interobserver variability of measurement of thrombus grayscale intensity in OCT images using image analysis software. Scatterplot and Bland-Altman plot of intraobserver (A) and interobserver (B) comparison for mean grayscale intensity. OCT, optical coherence tomography; SD, standard deviation.
(TIF)

S5 Fig Relationship of binary thrombus type and parameters measured by image analysis software in thrombus areas in OCT images. Scatterplots for median attenuation (A), 10^th^ percentile of attenuation (B), mean backscatter (C), 10^th^ percentile of backscatter (D), mean grayscale intensity (E) and 10^th^ percentile of grayscale intensity (F). OCT, optical coherence tomography.
(TIF)

S6 Fig Relationship of thrombus type and parameters measured by image analysis software in thrombus areas in OCT images using a higher, 0.5 mm^-1^ threshold for minimal attenuation. Scatterplots for median attenuation (A,D), 10^th^ percentile of attenuation (B,E) and 95^th^ percentile of attenuation (C,F). Thrombus attenuation score in the upper panel and bivariate thrombus type in the lower panel. OCT, optical coherence tomography.
(TIF)

S7 Fig Relationship of thrombus type and the ratio of 95^th^ percentile of attenuation and median grayscale intensity. Thrombus attenuation score (A) and binary thrombus type (B).
(TIF)
