# Supplementary figures and images for: Comparison of visual assessment and computer image analysis of intracoronary thrombus type by optical coherence tomography
Source: PLoS One. 2018 Dec 17;13(12):e0209110. doi: 10.1371/journal.pone.0209110 (PMC6296537; doi:10.1371/journal.pone.0209110)

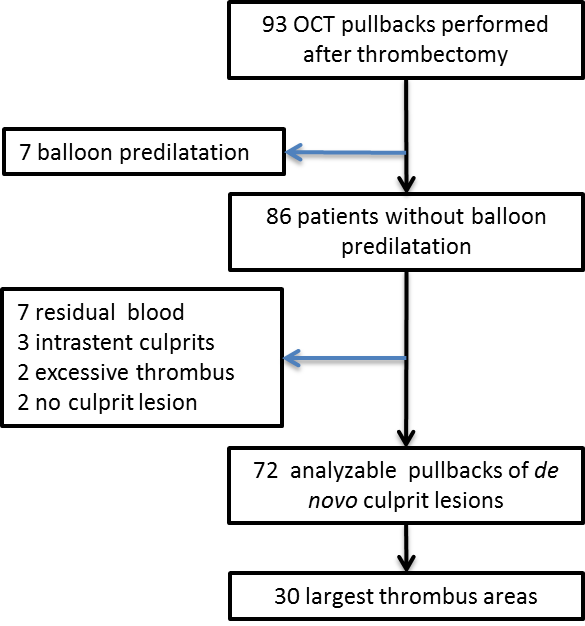

Supplement: S1 Fig — OCT, optical coherence tomography. (TIF) [file pone.0209110.s001.tif]

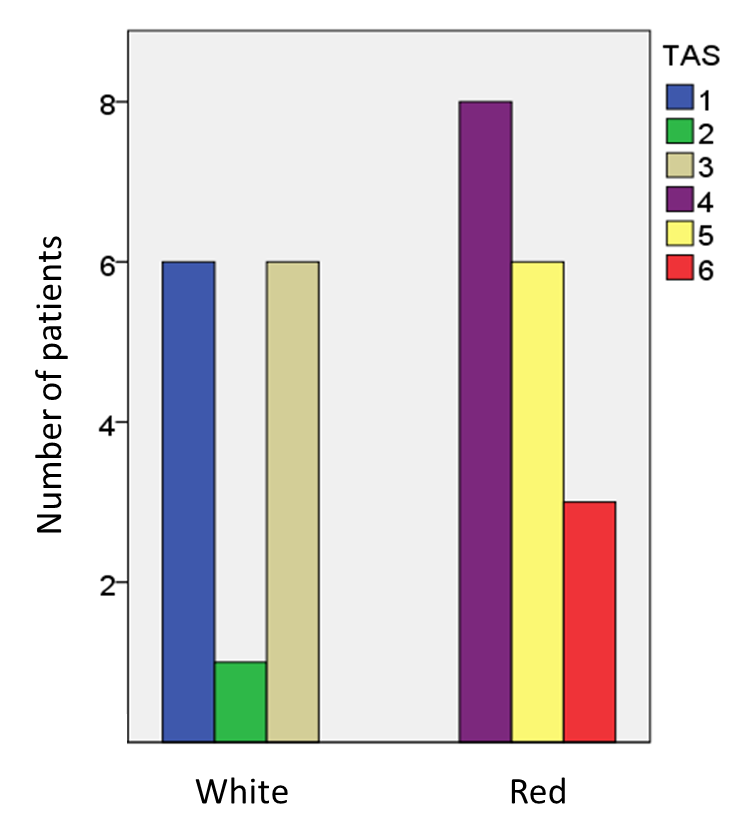

Supplement: S2 Fig — Consensus classification by two observers is shown. Thrombus attenuation score (TAS) from 1 to 3 equals white thrombus and from 4 to 6 red thrombus. (TIF) [file pone.0209110.s002.tif]

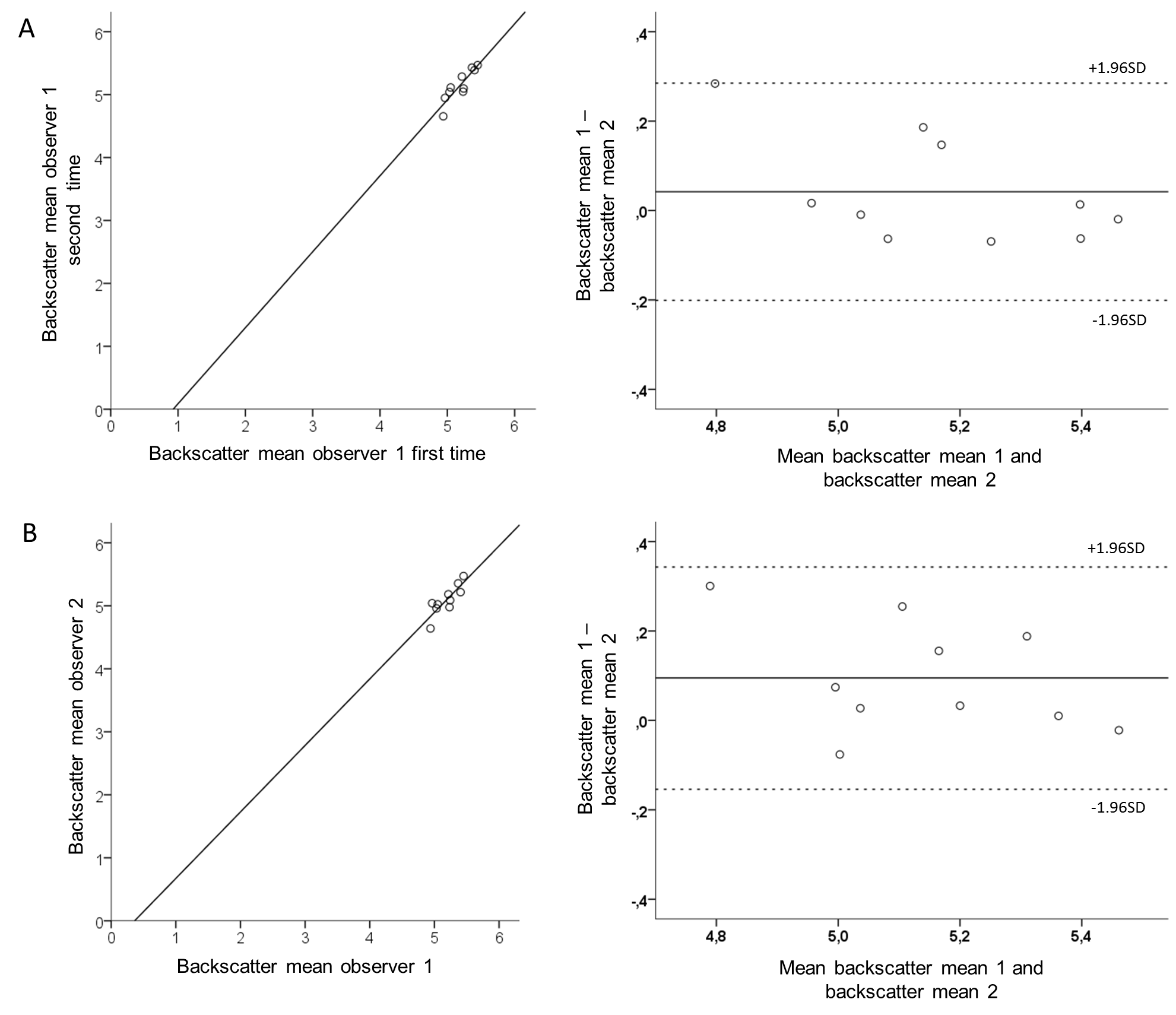

Supplement: S3 Fig — Scatterplot (left) and Bland-Altman plot (right) of intraobserver (A) and interobserver (B) comparison for mean backscatter. OCT, optical coherence tomography; SD, standard deviation. (TIF) [file pone.0209110.s003.tif]

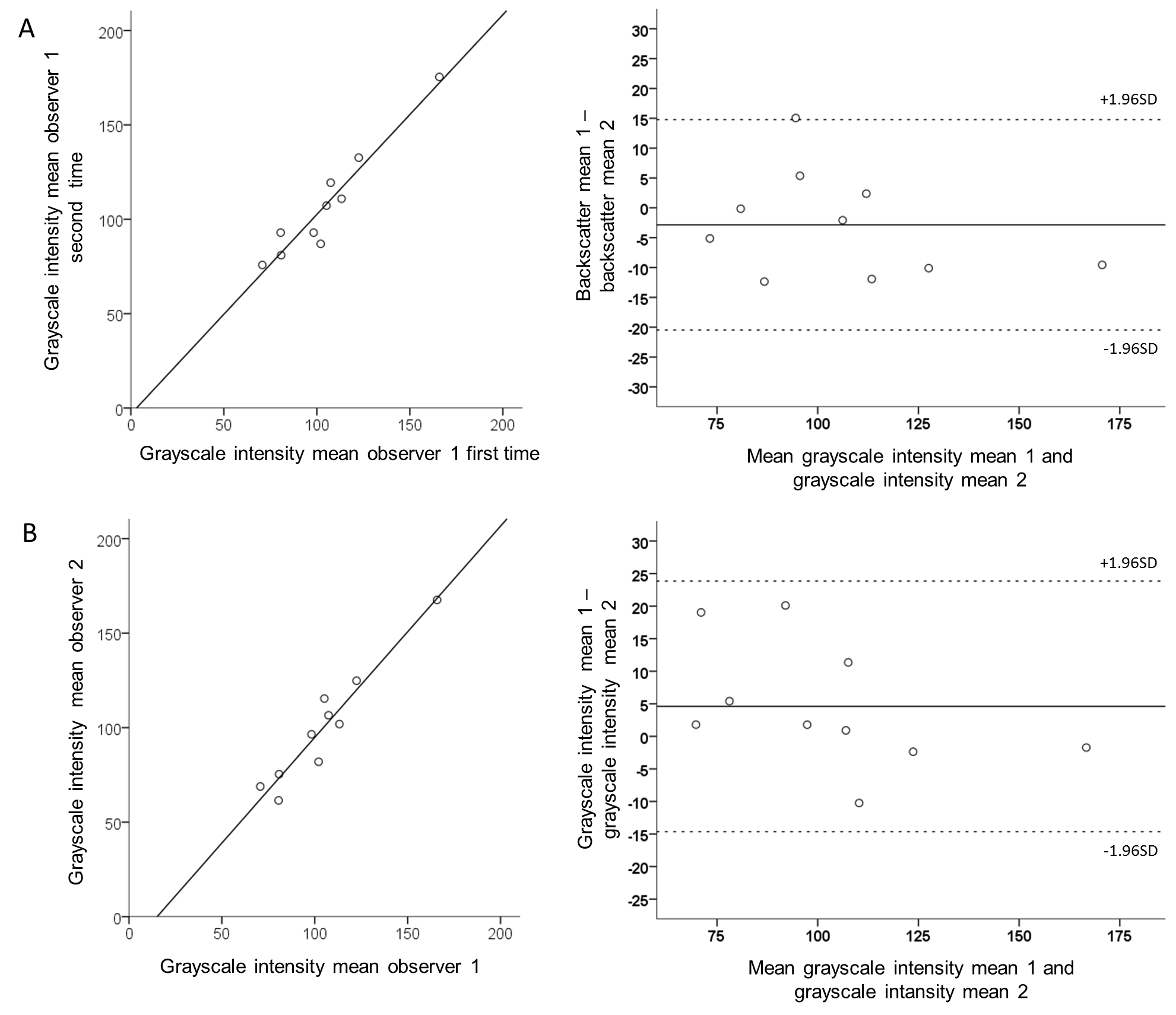

Supplement: S4 Fig — Scatterplot and Bland-Altman plot of intraobserver (A) and interobserver (B) comparison for mean grayscale intensity. OCT, optical coherence tomography; SD, standard deviation. (TIF) [file pone.0209110.s004.tif]

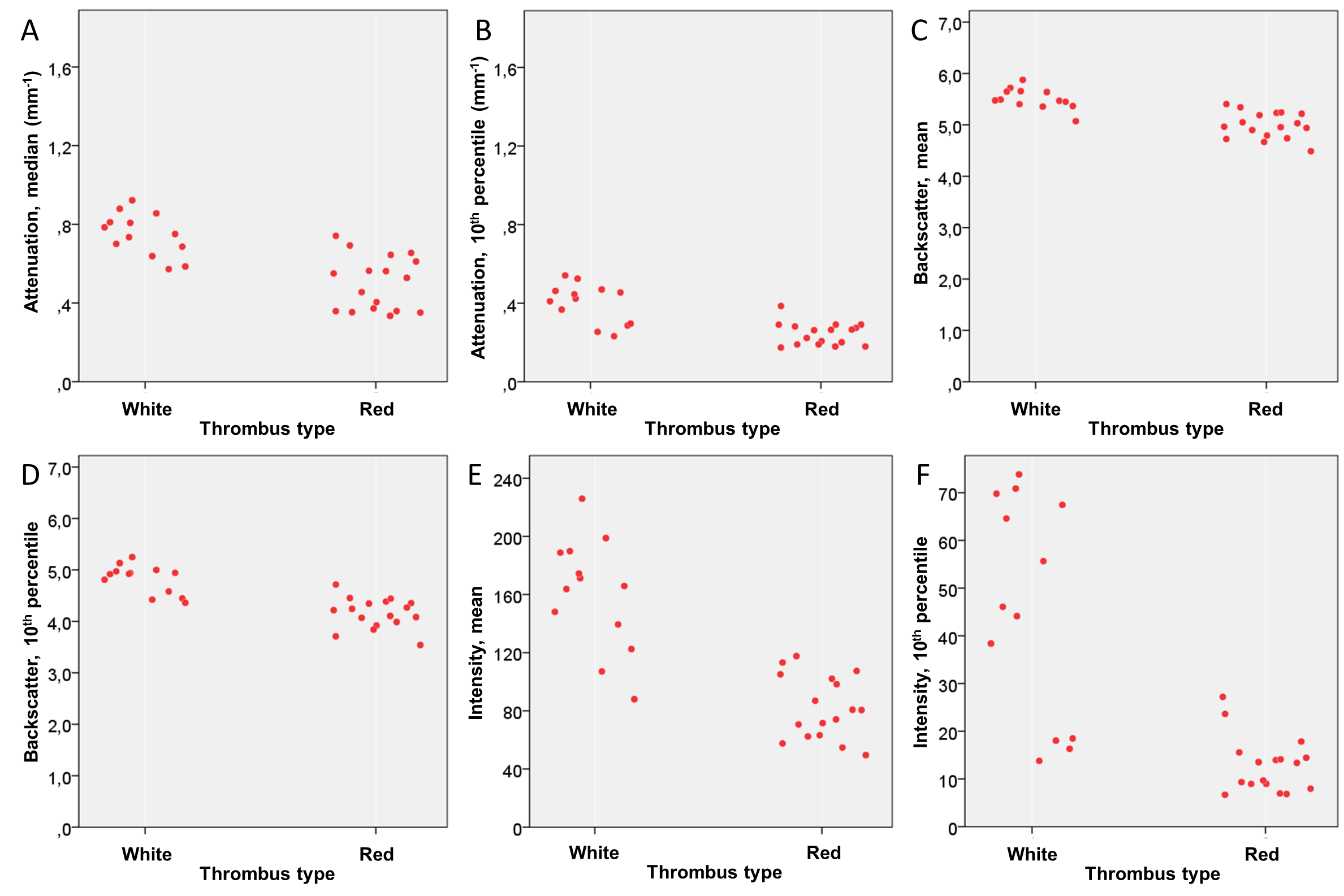

Supplement: S5 Fig — Scatterplots for median attenuation (A), 10th percentile of attenuation (B), mean backscatter (C), 10th percentile of backscatter (D), mean grayscale intensity (E) and 10th percentile of grayscale intensity (F). OCT, optical coherence tomography. (TIF) [file pone.0209110.s005.tif]

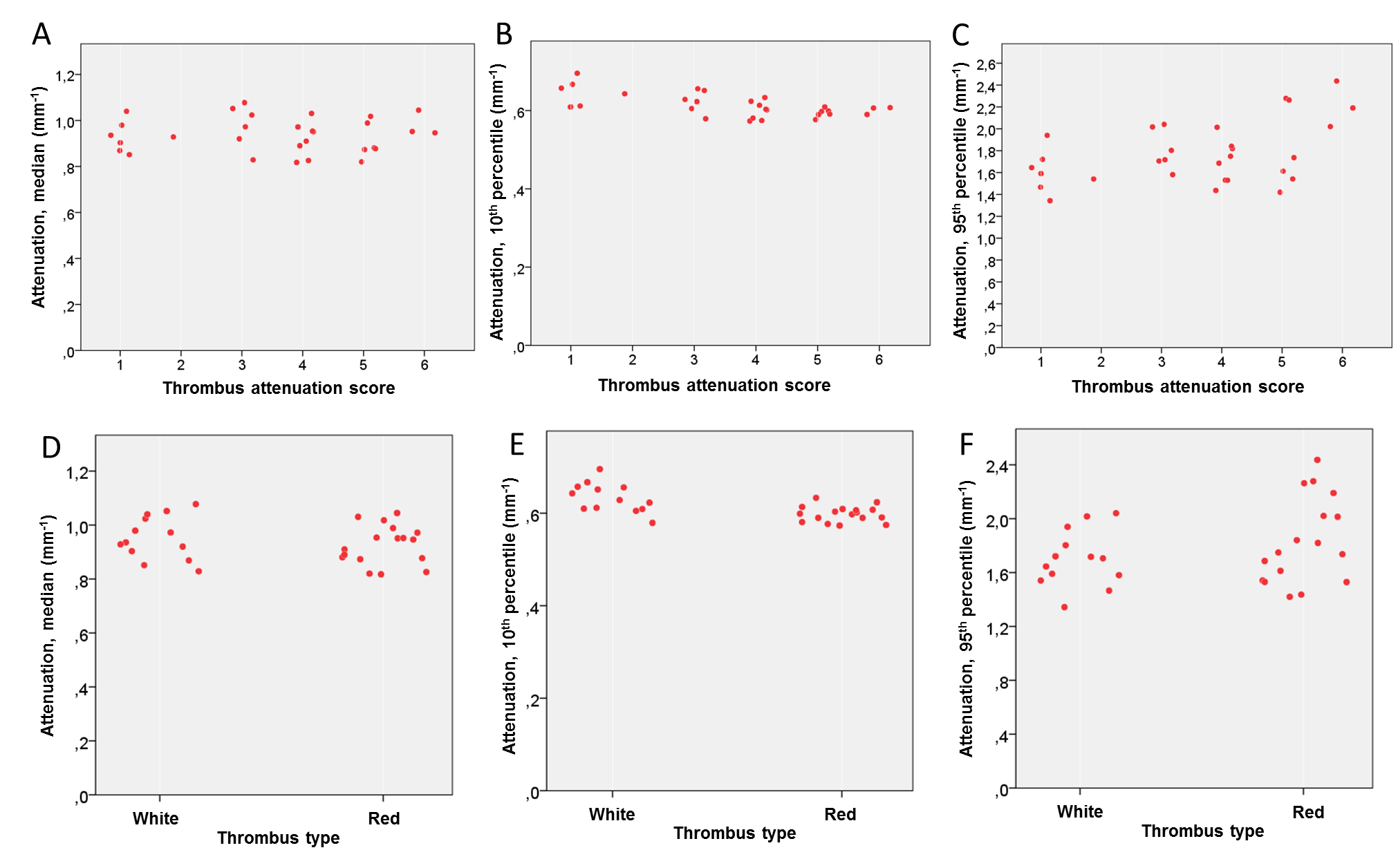

Supplement: S6 Fig — Scatterplots for median attenuation (A,D), 10th percentile of attenuation (B,E) and 95th percentile of attenuation (C,F). Thrombus attenuation score in the upper panel and bivariate thrombus type in the lower panel. OCT, optical coherence tomography. (TIF) [file pone.0209110.s006.tif]

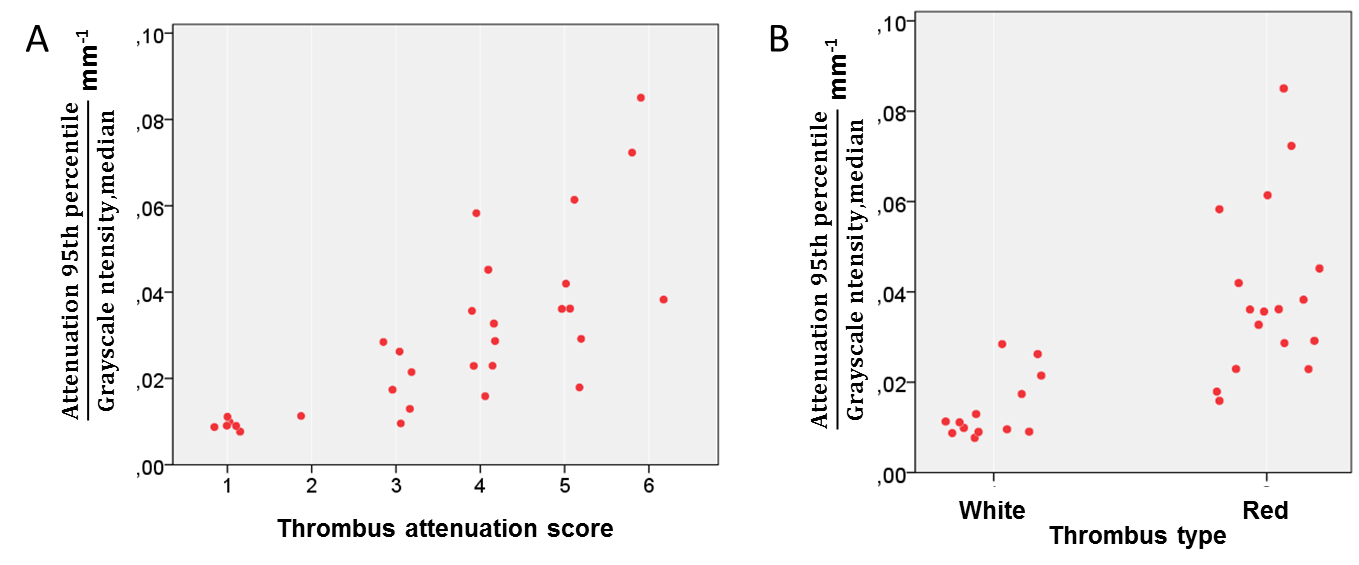

Supplement: S7 Fig — Thrombus attenuation score (A) and binary thrombus type (B). (TIF) [file pone.0209110.s007.tif]
